# Zika Virus Infection Leads to Variable Defects in Multiple Neurological Functions and Behaviors in Mice and Children

**DOI:** 10.1002/advs.201901996

**Published:** 2020-08-02

**Authors:** Ziqi Zhao, Ziwei Shang, Zilton Vasconcelos, Chunfeng Li, Yisheng Jiang, Shulong Zu, Jingyi Zhang, Fengchao Wang, Li Yao, Jae U. Jung, Patricia Brasil, Maria Elisabeth Moreira, Cheng‐Feng Qin, Tara Kerin, Karin Nielsen‐Saines, Genhong Cheng, Xiaohui Zhang, Zhiheng Xu

**Affiliations:** ^1^ State Key Laboratory of Molecular Developmental Biology CAS Center for Excellence in Brain Science and Intelligence Technology School of Future Technology Institute of Genetics and Developmental Biology Chinese Academy of Sciences Beijing 100101 China; ^2^ State Key Laboratory of Cognitive Neuroscience & Learning IDG/McGovern Institute for Brain Research Beijing Normal University Beijing 100875 China; ^3^ Instituto Fernandes Figueira Fundação Oswaldo Cruz (FIOCRUZ) Rio de Janeiro RJ 22250‐020 Brazil; ^4^ Institute for Immunity Transplantation, and Infection Stanford University Stanford CA 94305 USA; ^5^ Center for Systems Medicine Institute of Basic Medical Sciences Chinese Academy of Medical Sciences & Peking Union Medical College Beijing 100005 China; ^6^ Suzhou Institute of Systems Medicine Suzhou Jiangsu 215123 China; ^7^ Department of Molecular Microbiology and Immunology and Division of Maternal‐Fetal Medicine Department of Obstetrics and Gynecology Keck School of Medicine University of Southern California Zilkha Neurogenetic Institute Los Angeles CA 90033 USA; ^8^ Laboratório de Pesquisa Clínica em Doenças Febris Agudas Instituto Nacional de Infectologia Evandro Chagas Fondação Oswaldo Cruz (FIOCRUZ) Rio de Janeiro RJ 21040‐360 Brazil; ^9^ Department of Virology State Key Laboratory of Pathogen and Biosecurity Beijing Institute of Microbiology and Epidemiology Beijing 100071 China; ^10^ Division of Pediatric Infectious Diseases David Geffen School of Medicine UCLA Mattel Children's Hospital Los Angeles CA 90095 USA; ^11^ Department of Microbiology Immunology and Molecular Genetics University of California Los Angeles CA 90095 USA; ^12^ Parkinson's Disease Center Beijing Institute for Brain Disorders Beijing 100101 China

**Keywords:** abnormal behaviors, microcephaly, neurological functions, visual cortical functions, Zika virus

## Abstract

Zika virus (ZIKV) has evolved into a global health threat because of its causal link to congenital Zika syndrome. ZIKV infection of pregnant women may cause a spectrum of abnormalities in children. In the studies in Brazil, a large cohort of children with perinatal exposure to ZIKV is followed, and a spectrum of neurodevelopmental abnormalities is identified. In parallel, it is demonstrated that infection of the mouse neonatal brain by a contemporary ZIKV strain instead of an Asian ancestral strain can cause microcephaly and various abnormal neurological functions. These include defects in social interaction and depression, impaired learning and memory, in addition to severe motor defects, which are present in adult mice as well as in the prospective cohort of children. Importantly, although mouse brains infected later after birth do not have apparent abnormal brain structure, those mice still show significant impairments of visual cortical functions, circuit organization, and experience‐dependent plasticity. Thus, the study suggests that special attention should be paid to all children born to ZIKV infected mothers for screening of abnormal behaviors and sensory function during childhood.

## Graphical abstract

A spectrum of neurodevelopmental abnormalities is identified in children and neonatal mice with perinatal exposure to Zika virus (ZIKV), regardless of apparent abnormal brain structure. Thus, special attention should be paid to all children born to ZIKV infected mothers for screening of abnormal behaviors and sensory function during childhood.

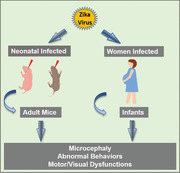

## 1 Introduction

The world's attention has been drawn to the global epidemic of Zika virus (ZIKV) infection in 2015–2016.^[^ ^1^, ^2^ ^]^ Contrary to the mostly mild and often asymptomatic phenotype identified in Africa and Asian countries in the past, contemporary ZIKV causes defects in developing fetuses and neonates born to mothers infected during pregnancy. The most severe manifestation is recognized as congenital Zika syndrome and includes microcephaly and other serious congenital neurological complications.^[^ ^1^, ^2^, ^3^, ^4^ ^]^ Those are mostly due to the ZIKV infection during the first or second trimester of pregnancy. Many of the children with microcephaly at birth have severe motor impairment, vision and hearing abnormalities, language disability, seizure disorders, and sleep difficulties by age two years.^[^ ^5^, ^6^, ^7^, ^8^, ^9^ ^]^ However, the long‐term effects of perinatal exposure to ZIKV, including the expected abnormal behaviors, are still not clear.

Several animal models have been established to show the links between ZIKV infection and microcephaly and associated neurological abnormalities.^[^ ^10^, ^11^, ^12^, ^13^, ^14^ ^]^ Since infection of fetal or neonatal mice lead to their death shortly after birth, thereafter, most studies of ZIKV infection in the fetal brain have been focused on its short‐term effects.^[^ ^10^, ^11^, ^12^, ^13^ ^]^ In addition, whether ZIKV infection during the third trimester of pregnancy, which usually did not cause microcephaly, has any damaging impact is not very clear. Therefore, long‐term effects of ZIKV infection, especially the abnormal neurological functions and behaviors in both mice and humans need to be explored in order to provide a guideline for the early identification of potential disabilities in affected children.

## 2 Results

**Figure 1:**
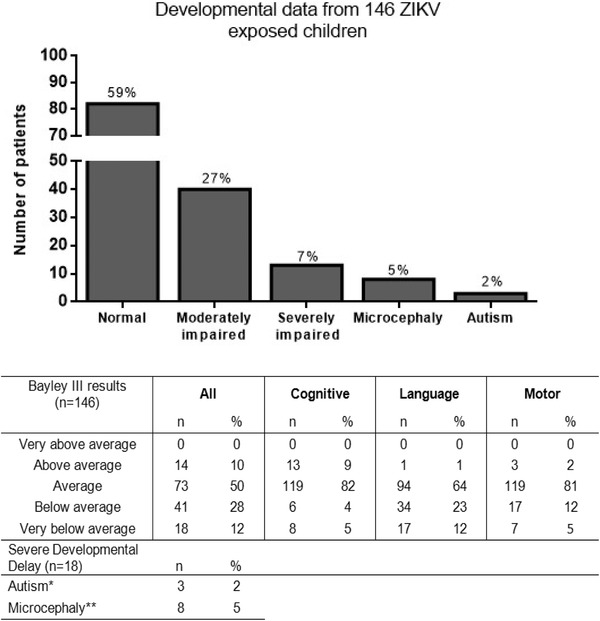
Bayley III analysis of 146 children with antenatal ZIKV infection. Range of developmental abnormalities among 146 children who had developmental testing performed. Among those with severe impairment, 8 had microcephaly and 3 had autism spectrum disorder while 8 children did not have microcephaly nor ASD, for a total of 12% severely impaired children with neurodevelopmental testing performed between 7 to 32 months of age.

In parallel to the mouse model studies, we evaluated a large cohort of 146 children with antenatal ZIKV infection prospectively in Rio de Janeiro, Brazil since the time of maternal ZIKV acute infection during pregnancy.^[^ ^9^, ^15^, ^16^, ^17^ ^]^ At an older age range between 12 to 32 months, these children who were born during and shortly after the ZIKV epidemic had a spectrum of neurodevelopmental abnormalities which went from normal to severely impaired.^[17]^ Bayley III neurodevelopmental testing in the second and third years of life in 146 children within our cohort demonstrated skewed results.^[17]^ No children demonstrated high neurodevelopmental functioning (>2 × SD in any Bayley III score result, Table S1, Supporting Information) in any of the three neurodevelopmental domains (cognitive, language or motor).^[^ ^16^, ^17^ ^]^ Below average functioning, consistent with moderate to severe neurodevelopmental impairment (−1 to <−2 × SD results for Bayley III scores) in any of the functional domains was noted in over 30% of children while above average functioning (1 to 2 × SD) was only seen in 10% of children in any of the three neurodevelopmental domains (**Figure** **1**). Among 12% of children with severe neurodevelopmental impairment, microcephaly, and/or autism was seen in over half of the children. Further analyses demonstrated that the receptive language component is more affected than the expressive component in our population of children. We believe the abnormalities noted likely result from ZIKV‐mediated direct brain injury as opposed to lack of infant stimulus.

**Figure 2:**
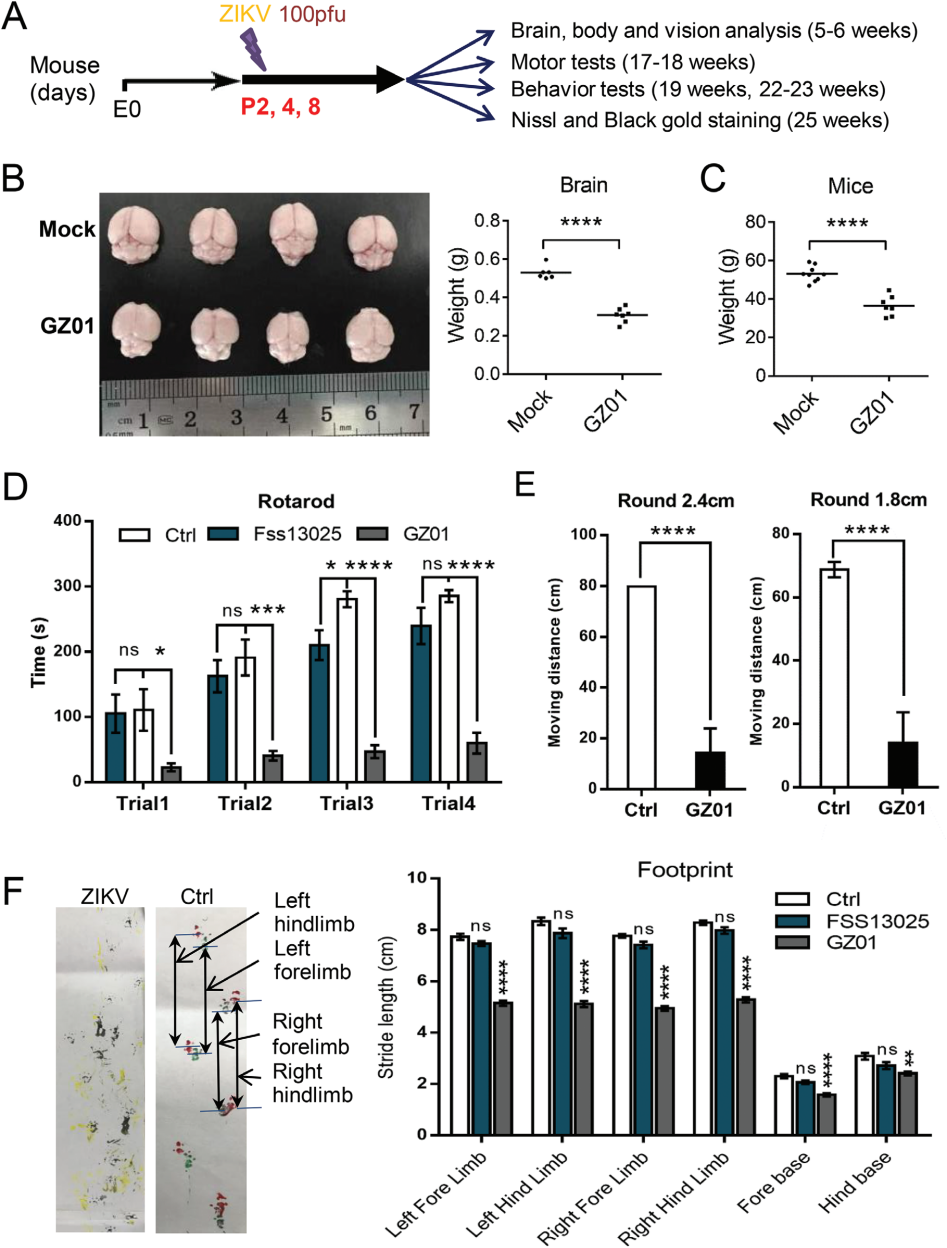
Neonatal GZ01 ZIKV infection leads to much more severe motor defects than FSS13025 ZIKV in mice. A) Timeline for ZIKV inoculation and the monitoring of a complementary set of clinical, virological, and neuropathological outcomes from postnatal day 2 (P2) to adult. All mice were infected with ZIKV (GZ01 unless specified, 100 PFU per mouse) or DMEM intracranially at P2. B) Weight of brain and C) whole body were inspected at 6 weeks. D) The latency to fall off from the Rotarod during the four trials for each mouse at 18 weeks. (*n*Ctrl = 6 mice, *n*Fss13025 = 8 mice, *n*GZ01 = 8 mice). E) The distance for each mouse traveled on round beams in different diameters at 14 weeks (*n*Ctrl = 10 mice, *n*GZ01 = 8 mice). F) Representative paw print at 25 weeks. The data of all the stride lengths. (*n*Ctrl = 6 mice, *n*Fss 13025 = 8 mice, *n*GZ01 = 8 mice) All the data shown are mean ± SEM. ^****^ *p* < 0.0001, ^***^ *p* < 0.001, ^**^ *p* < 0.01, *p < 0.05, ns *p* > 0.05, *t*‐test. For each group, *n* ≥ 8.

Since a spectrum of neurodevelopmental abnormalities were found in our prospective cohort of children, we proposed to establish a perinatal ZIKV infected mouse model that could survive for a long period of time. 100 pfu of ZIKV FSS13025 (an ancestral Asian strain isolated in Cambodia in 2010)^[18]^ or GZ01 (a contemporary Asian strain isolated from a patient infected in Venezuela in 2016)^[19]^ or Dulbecco's Modified Eagle Medium (DMEM) was injected into the *λ* point of ICR or C57 mouse brains started at postnatal day 2 (P2) (**Figure** **2**A). Mouse brain at P2 equates to the beginning of the 3rd trimester in humans.^[20]^ Half of the mouse population infected with GZ01 can survive for nearly a year. Smaller brain size and body weight were detected at 6 weeks (Figure 2B,C). We inspected potential effects of ZIKV infection in grownup mice as highlighted in Figure 2A. Considering that most children with ZIKV‐associated microcephaly have severe motor impairment noted shortly after birth^[^ ^5^, ^6^, ^7^, ^8^, ^9^ ^]^ and the disorder was also detected in many children in our cohort, we evaluated mice at postnatal 17–18 weeks with the Rotarod test. GZ01‐infected mice showed a dramatic decrease in latency to fall when compared to FSS13025‐infected and mock‐infected animals (controls) (Figure 2D). The motor dysfunction of GZ01‐infected mice was also manifested in beam walking tests on different sizes of square sticks or round sticks (Figure 2E; Figure S2, Supporting Information). Coordinated distal motor function was further inspected by footprint analysis. As shown in Figure 2F, GZ01‐infected animals moved clumsily, and their stride lengths of all four limbs, and both fore and hind bases were significantly shorter compared with the two other groups. These results indicate that the motor function of GZ01 instead of FSS13025‐infected mice was severely impaired, which is consistent with our findings observed in perinatally infected children.

**Figure 3:**
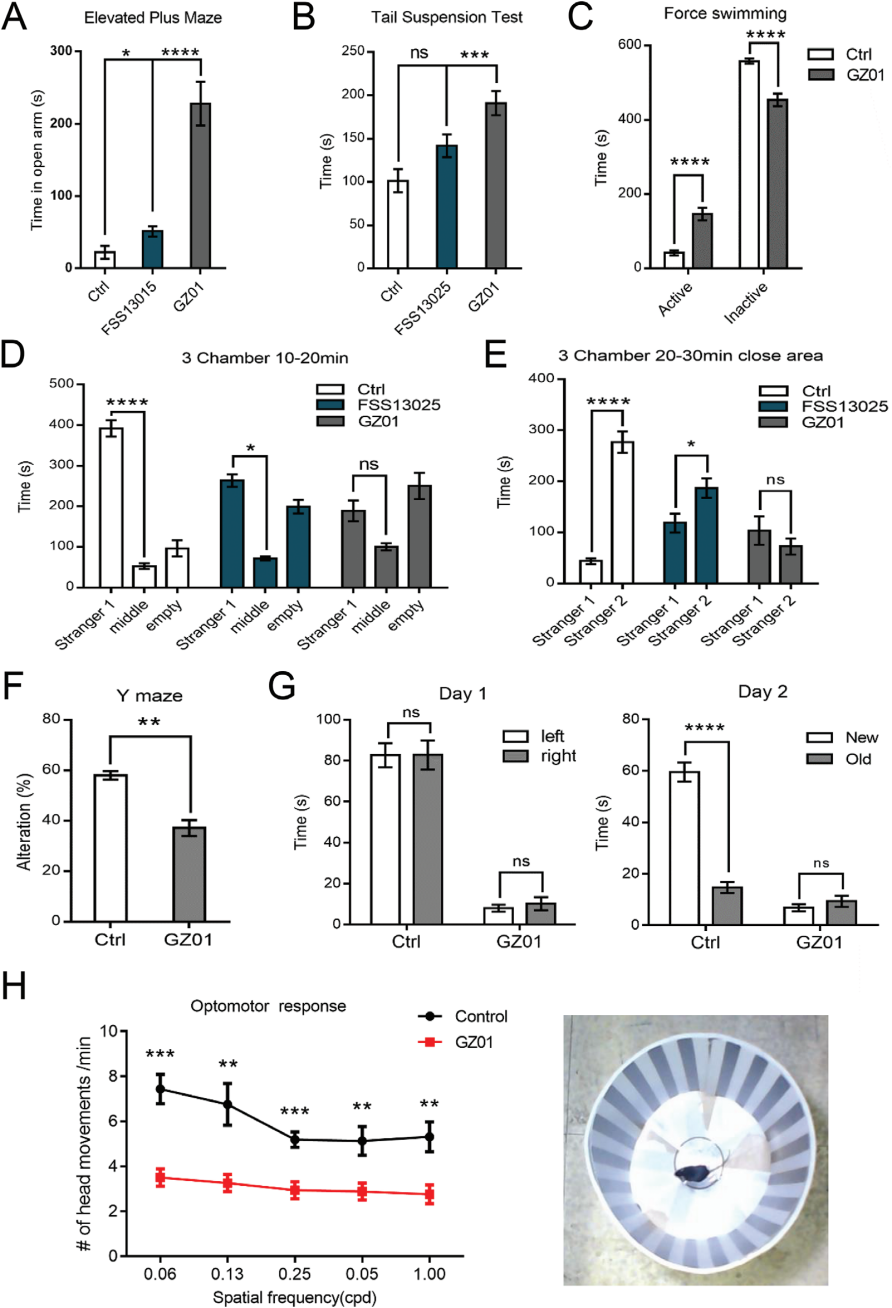
Neonatal ZIKV infection leads to abnormal behaviors and visual defect in mice. A) The time spent in open arms for each group of control, FSS13025 or GZ01 ZIKV infected mice during the elevated plus maze test at 13 weeks. B) The active time of mouse during tail suspension test at 16 weeks. C) The active and inactive time of mice in force swimming test at 12 weeks. D,E) The time of mice spent on socializing with Stranger 1 and Stranger 2 during the 3‐chamber test at 23 weeks. F) The ratio of alteration of each mouse in Y maze test at 22 weeks. G) The time of mouse spent observing novel and old objective on day1 and day 2 respectively at 19 weeks. H) Statistics of the number of head movements/min of control and GZ01 P4‐infected mice during optomotor responses tests. Photograph of mouse in the rotating drum covered with black and white stripes at a spatial frequency of 0.06 cpd. In (A,B,D,E) *n*Ctrl = 6 mice, *n*FSS13025 = 8 mice, *n*GZ01 = 8 mice. In (C,F,G) *n*Ctrl = 6 mice, *n*GZ01 = 8 mice. In (H) *n* = 8 per group. All the data showed mean ± SEM, ^****^ *p* < 0.0001, ^***^ *p* < 0.001, ^**^ *p* < 0.01, *p < 0.05, ns *p* > 0.05.

Because abnormal behaviors were detected in perinatally infected children, we sought to investigate whether the GZ01 infected‐mice (aged at 22–23 weeks) would have behavioral impairments using a battery of behavioral tests. Interestingly, in the elevated plus maze tests, GZ01‐infected mice spent substantially longer time in the open arms compared with FSS13025‐infected mice and controls (**Figure** **3**A). In both force swimming and tail suspension tests, we observed a significant increase of active time of GZ01‐infected mice (Figure 3B,C). We further used the three‐chamber test to inspect animals for their voluntary initiation of social interaction and their preference for social novelty. In the sociability phase, control mice spent significantly more time in the chamber with the social partner (Stranger 1) than the empty cage. In contrast, GZ01‐infected mice showed no apparent preference while the FSS13025‐infected mice spent just a little more time with the social partner (Figure 3D). In the social novelty phase, a novel partner (Stranger 2) was introduced into the previously empty cage. Control mice displayed a preference for Stranger 2, while GZ01 infected mice showed again no apparent preference and FSS13025‐infected mice displayed little preference (Figure 3E). These results indicated the GZ01‐infected mice exhibited significant defects in social interaction behaviors and reduced levels of anxiety and depression, while FSS13025‐infected mice had much less abnormal behaviors.

Y maze and novel object recognition tests were performed to determine whether infected mice had defects in learning and memory function. In the Y maze test, GZ01‐infected mice showed similar number of total arm entries but apparent reduction in spontaneous alternation (Figure 3F), suggesting a severe deficit in their spatial working memory.^[21]^ During the object recognition test, all mice did not have a preference for the toy on either the left or right side on day 1, while GZ01‐infected mice spent much less time with toys (Figure 3G). On day 2, control mice spent more time with the new toy while GZ01‐infected mice did not show any preference and spent much less time with the toys as well (Figure 3G). Thus, GZ01‐infected mice are likely to have poor spatial working memory and a defective hippocampus‐dependent pattern recognition function.

Since vision defect was detected in perinatally infected children,^[^ ^5^, ^6^, ^7^, ^8^, ^9^ ^]^ we infected C57 pups at P4 with GZ01 and performed optomotor response test at P30. Infected mice showed significantly reduced numbers of head movements at all five frequencies in a motorized drum (Figure 3H). This demonstrated that ZIKV infection at P4 led to severe deficit of vision in mice.

After completion of behavioral tests, we inspected the mouse brain structure at 25 weeks of age. Mice infected with GZ01 at P2 had substantially smaller brain sizes and thinner cortex than controls (Figure S2A, Supporting Information). The histocytochemical results showed apparent neuron loss and swelling in the infected cortex (Figure S2A, Supporting Information). In addition, both the thickness and ratio of myelination in infected brains were significantly reduced (Figure S2B, Supporting Information). Significant activation of the astrocytes was also detected in the prefrontal cortex which moderates social behaviors (Figure S2C, Supporting Information). These findings indicated that neonatal brain infection with GZ01 led to long‐lasting brain degeneration which led to loss of neurons and defective myelination.

Because most of the perinatally infected children did not show significant microcephaly (Figure 1), we chose to infect mouse brains with GZ01 at P8 when they matched the end of the 3rd trimester in humans.^[20]^ Heat‐inactivated ZIKV (Mock) was used as controls. We evaluated the motor function of those mice. The latency to fall off from the Rotarod showed no difference between two groups (Figure S3A, Supporting Information). Similarly, infected mice managed to walk through the elevated narrow beam (Figure S3B, Supporting Information). We then performed footprint analysis and found most of the four limbs stride lengths of infected mice were similar to those of controls, except the left forelimbs (Figure S3C, Supporting Information). This indicated that motor function of those mice was not significantly impacted. We also performed the three‐chamber social interaction test. In the sociability phase, infected mice showed significantly weaker desire to contact with the social partner and displayed just a little preference for the novel partner (Figure S4, Supporting Information). This demonstrated that infection at P8 also caused defects in social interaction behaviors, although not as severe as infection at P2 (Figure 3D,E).

**Figure 4:**
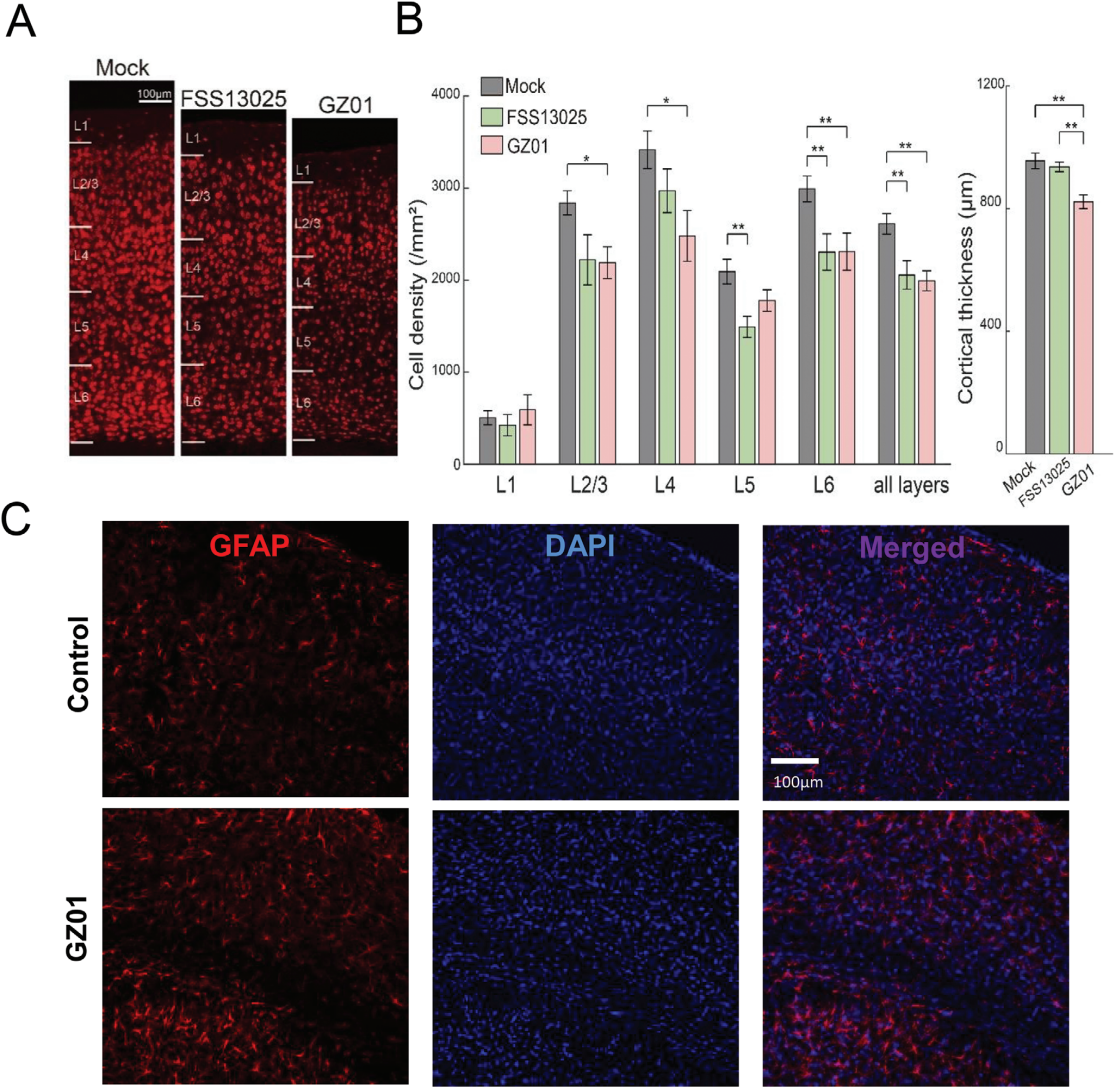
Neuronal loss and astrocyte activation in the visual cortex of ZIKV P8‐infected mice. A) NeuN (red) immunostaining images of the primary visual cortex of the GZ01, FSS13205 and Mock mice. B) Left: Statistical results of the cell density of individual layers and the overall density in the V1 of the Mock, FSS13205, and GZ01 mice, respectively. Right: Comparison of the mean values of cortical thickness among the three groups. *p < 0.05; ^**^ *p* < 0.01; Student *t*‐test. *n* = 6 sections from three juvenile mice for all three groups. C) GFAP (red) and DAPI (blue) staining (aged 4 months) of the primary visual cortex.

We went on to investigate the potential visual deficit in P8 infected mice. Histological assay of neural structures of the central visual pathways from the retina to the dorsal lateral geniculate body (dLGN) and V1 in the P28‐30 brain suggested that FSS13025 or GZ01 did not cause significant changes in the size and gross structure of these major vision‐related areas, except that the V1 thickness was reduced by ≈13% due to a small loss of neurons in individual cortical layers in the GZ01‐infected mice and the activation of astrocytes was detected (**Figure** **4**A–C; Figure S5A,B, Supporting Information) In addition, retrograde‐tracing analysis with retrovirus suggested that the axonal projection into the visual cortex was not affected significantly (Figure S5C, Supporting Information).

**Figure 5:**
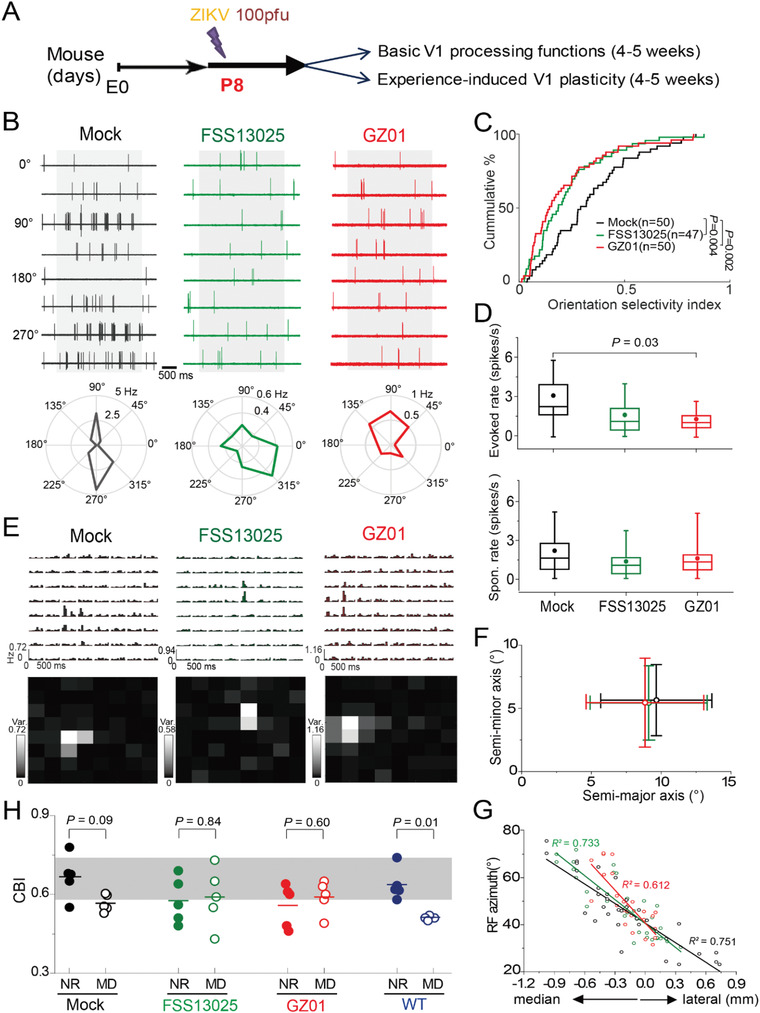
Neonatal infection of ZIKV impairs visual cortical functions and plasticity in juvenile mice. A) Time line of ZIKV infection and in vivo assay of visual cortical functions and plasticity. B) Raster (top) and polar plots of spikes evoked by drifting gratings with 8 orientations of the example V1 cell recorded in the Mock, FSS13025 and GZ01 mice, respectively. C) Reduced orientation tuning function of V1 in the FSS13025 and GZ01 groups indicated by the cumulative percentage distribution of orientation selectivity indices. *p* values were calculated by the Kolmogorov–Smirnov test. D) Comparisons of spike rates of visually evoked (top) and spontaneous baseline (bottom) activity of V1 cells among the 3 groups. *p* values were calculated by the unpaired *t*‐test. E) Representative RFs of the V1 cells in the 3 groups, manifested by both PHSTs of spike responses to flashing squares (in 10°) in individual grid positions (top) and the calculated spatial RFs based on spiking response variances (bottom). F) Little difference in the mean RF size of V1 cells among the three groups (one‐way ANOVA test). G) Compressed retinotopic maps in the V1 of both FSS13025 and GZ01 groups in comparison to the Mock, manifested by plots of V1 cells RF central azimuths relative to the vertical meridian (medial–lateral positions). Note that the 0 mm position corresponds to the visual field at 40° in each animal. H) Plots of the CBI value measured from each animal that were either normally reared (solid) or experienced 4‐day MD in each group (Mock, FSS13025, GZ01 and wild type WT). Shadow indicates the normal CBI range of the WT animals. *p* values were calculated by the Mann–Whitney test.

We conducted in vivo electrophysiological recording in the primary visual cortex (V1) at the juvenile age to directly examine sensory circuit function and plasticity (**Figure** **5**A). We recorded single‐unit spiking activity of visual cortical neurons in the V1 binocular zone with glass micropipettes at P29–31 when specific visual stimuli were presented to each eye of anesthetized animals (see the Experimental Section in the Supporting Information). Normally mouse V1 neurons show their spiking activity preferences to specific spatiotemporal features of visual inputs, known as neuronal tuning functions including the orientation selectivity (OS) and the receptive field (RF).^[^ ^22^, ^23^, ^24^ ^]^ The drifting gratings of eight different orientations were used to assay the OS of individual V1 neurons by measuring evoked spike‐rates at corresponding orientations in the Mock, FSS13025, or GZ01 mice, respectively (Figure 5B). In population, V1 neurons in the GZ01 and FSS13025 mice showed substantially reduced OS values, where OS values of the GZ01 group were more severely reduced compared to that of the Mock group (Figure 5C; *p* = 0.004, FSS13025 vs Mock; *p* = 0.002, GZ01 vs Mock, Kolmogorov–Smirnov test). A relatively bigger reduction in the mean rate of evoked spiking‐responses (measured at optimal orientations of individual neurons) in the GZ01‐infected mice could account for the smaller OS values (Figure 5D top; *p* = 0.03, GZ01 vs Mock, Kolmogorov–Smirnov test), while no difference was observed in the mean baseline spike rate among the three groups (Figure 5D bottom). Remarkably, the V1 of GZ01‐infected mice had a severely compressed visual‐field map (retinotopy) in comparison with that of the FSS13025 or Mock mice (Figure 5G), although the RF size of visual cortical cells for the three groups did not show significant difference, assayed by the spare‐noise flashing squares of 8 × 8 grids, (Figure 5E,F). Together, these in vivo recording results suggest that neonatal brain infection of FSS13025 or GZ01 at P8 impairs a normal development of visual functions and retinotopic map organization of the V1 in juvenile mice, and more deteriorating effects are evident in GZ01‐infected animals.

Maturations of visual cortical function and organization are critically dependent on experience‐induced neural circuit plasticity during a critical juvenile period.^[^ ^25^, ^26^ ^]^ Thus, we next tested whether neonatal infection of ZIKV might affect the critical period V1 plasticity in juvenile mice, by examining the classical monocular deprivation (MD)‐induced ocular dominance (OD) plasticity of juvenile V1. OD preferences of the population V1 cells in the contralateral binocular zone were assayed by in vivo measuring their spike responses to drifting gratings presented to each eye after 4‐day MD to one eye (by eyelid suture) during P26–29.^[^ ^22^, ^27^, ^28^ ^]^ The MD‐induced OD shifts of tested binocular V1 cells were depicted by the classical 7‐scale histogram of OD by grouping ODIs into seven categories (the left two columns in Figure S6, Supporting Information) and the resultant calculated contralateral bias index (CBI) for tested individual animals (Figure 5H, see the Experimental Section in the Supporting Information). Our results showed that mice injected with heat‐inactivated Mock ZIKV, 4‐day MD caused an apparent shift of V1 binocular cell responses toward the undeprived ipsilateral eye, measured by the ocular dominance index (ODI; top right Figure S6A, Supporting Information, *p* = 0.04), similar to that found in the wild‐type mice (Figure 5H). In contrast, same MD treatment to the FSS13025 or GZ01 mice did not induce any shifts in the OD distribution of V1 cells (Figure 5H; middle and bottom rows of Figure S6, Supporting Information; *p* = 0.60 for the FSS13025; *p* = 0.24 for the GZ01, Kolmogorov–Smirnov test). Taken together, these results indicate that the neonatal brain infection of ZIKV at P8 also severely disrupts the early experience‐dependent plasticity of sensory cortical circuits in juvenile mice.

## 3 Discussion

Prompted by the findings observed in a large cohort of children followed longitudinally, we developed an animal model to further investigate this phenomenon. In the children with antenatal ZIKV exposure, over 30% had moderate to severe neurodevelopmental impairment. In children with severe neurodevelopmental impairment, microcephaly and/or autism was seen in over half of the group.^[17]^ To mimic the clinical perinatal ZIKV infection, we infected newborn mice with ZIKV at mainly two time points and systemically evaluated the long‐term consequences of infection. In P2 infected mice, the contemporary American strain (GZ01) and, to a much less degree, ancestral Asian strain (FSS13025) caused various apparent abnormal brain structures and functions as well as behaviors that were reported in patients. Importantly, our findings also indicate that those mice also have abnormal social behaviors which are consistent with the findings in our cohort. However, the reduction of anxiety and depression levels is not yet reported clinically. We may predict that children with ZIKV associated microcephaly will show those abnormalities as they grow up. Moreover, in mice infected at P8, no significant abnormality in cellular structures and axonal projection along the central visual pathway was detected. However, this later infection of ZIKV can lead to significant impairments of visual functions, retinotopic organization, and experience‐dependent cortical plasticity in juvenile V1. Especially, the early experience‐dependent plasticity of juvenile brain is critical for the formation of precise neuronal connections and normal circuitry functions in many brain circuits.^[^ ^25^, ^26^, ^29^, ^30^ ^]^ In addition, less severe abnormal social behaviors were also detected in mice infected at P8. We thus speculate that these neuropathological defects of neuronal circuit functions and plasticity can also importantly account for defects of various behaviors and mental functions. According to the previous and current mouse models,^[^ ^10^, ^11^, ^12^, ^13^, ^14^ ^]^ early brain infection (before embryonic 13.5 day, E13.5) leads to miscarriage while infection at the middle to later embryonic and the neonatal stages (E13.5‐P3) would cause microcephaly. Postnatal infection at P8 would not cause significant microcephaly but still incurs some abnormal brain functions. Whether the variable defects in cognitive, language, social interaction and motor present in our cohort of children are due to the timing of ZIKV infection is currently undergoing inspection.

Our mouse findings are consistent with those noted in children antenatally exposed to ZIKV which demonstrates translatability of our mouse model to humans. A similar pattern was observed in our animals infected at early postnatal stage and that of children with perinatal exposure to ZIKV including microcephaly, severe motor impairment, neurocognitive disorders, and abnormal social behaviors. These phenotypes are likely due to the long‐lasting degeneration of neurons and activation of astrocytes which leads to loss of neurons and defective myelination in the long‐term. Our finding that animals infected at both early and later postnatal stage leads to significant impairments of visual functions and experience‐dependent cortical plasticity fortifies the notion of underestimated magnitude of the consequences of the ZIKV epidemics and long‐term studies of developmental outcomes in children with congenital or perinatal ZIKV infection are needed. Thus, our animal study strongly mimics human data and suggests that attention should be paid to all children born to ZIKV infected mothers or children infected around the time of birth, regardless of the presence of microcephaly, with special attention to abnormal behaviors and vision needed during their childhood. Hopefully, this study will help in providing a guideline for the early identification of potential disabilities that will enable early intervention and improvement of the life quality of affected children.

## Conflict of Interest

The authors declare no conflict of interest.

## Authors Contributions

Z.Z., Z.S., Z.V., and C.L. contributed equally to this work. Z.X., X.Z., C.L., K.N.‐S and G.C. jointly directed the research, designed the experiments, and wrote the manuscript. Z.Z, Z.S., Z.V., and C.L. designed and performed most of the experiments. Y.J., S.Z., J.Z., F.W., and L.Y., helped in performing the experiments. J.U.J, P.B., M.E.M., C.‐F.Q., and T.K. helped in revising the manuscript and providing the critical reagents.

## Supporting information

Supporting Information
