# Supplementary material for: Zika Virus Infection Leads to Variable Defects in Multiple Neurological Functions and Behaviors in Mice and Children
Source: Adv Sci (Weinh). 2020 Aug 2;7(18):1901996. doi: 10.1002/advs.201901996 (PMC7509663; doi:10.1002/advs.201901996)
Supplement: Supplementary file 1 — Supporting Information [file ADVS-7-1901996-s001.pdf]

WILEY-VCH

© 2020 Wiley-VCH GmbH

ADVANCED SCIENCE Open Access

Supporting Information

for *Adv. Sci.,* DOI: 10.1002/advs.202001996

Energy Efficient Control of Ultrafast Spin Polarized Current to Induce Single Femtosecond Pulse Switching of a Ferromagnet

*Quentin Remy, Junta Igarashi, Satoshi Iihama, Grégory Malinowski, Michel Hehn, Jon Gorchon, Julius Hohlfeld, Shunsuke Fukami, Hideo Ohno, and Stéphane Mangin**

WILEY-VCH

© 2020 Wiley-VCH GmbH

Supporting Information

Energy Efficient Control of Ultrafast Spin Polarized Current to Induce Single Femtosecond Pulse Switching of a Ferromagnet

*Quentin Remy, Junta Igarashi, Satoshi Iihama, Grégory Malinowski, Michel Hehn, Jon Gorchon, Julius Hohlfeld, Shunsuke Fukami, Hideo Ohno and Stéphane Mangin**

S1. Hysteresis loops

Magnetic hysteresis loops were measured using the Magneto Optical Kerr Effect with a He-Ne continuous laser (633 nm) in a polar configuration. A photoelastic modulator combined with a lock-in amplifier were used to increase the signal to noise ratio.

S2. Femtosecond laser

A standard femtosecond laser shooting pulses of duration around 35 fs at a repetition rate of 5 kHz with a wavelength of 800 nm corresponding to a photon energy of 1.55 eV was used. The Gaussian beam diameter was determined in two ways: by directly observing the beam at the focal plane of the microscope's lens (used for imaging) and by using the domain size vs pulse energy fit (see Data analysis). Both methods provided consistent results and were used to determine the uncertainty of the beam size. Laser beam diameters measured for both spin valve samples are shown in **Table S1**. The power of the laser beam was controlled using a polarizer with a half-wave plate. The beam had a normal incidence with respect to the sample.

S3. MOKE microscope imaging

A LED (center wavelength around 630 nm) is used as the light source. For each laser power and each magnetic configurations, a picture of the sample was taken, being used as the background image in the data analysis. Other pictures were taken after each laser shot, without moving the sample. All pictures were averaged 16 times. Laser excitation was done on one side of the sample and MOKE microscope observation on the other. A magnetic field was applied to reach the desired magnetic configuration but it was removed during laser excitation. Only the MOKE images after the first two pulses are shown since the results after an odd (even) number of pulses is qualitatively identical to the result after the first (second) pulse.

S4. Data analysis

For each laser pulse shot, the background image was subtracted to the raw image. The magnetic domains diameters were estimated (the uncertainty is found from the spatial profile of the domain). The contrast was adjusted so that the maximum (minimum) displayed pixel value is the one that corresponds to the magnetic configuration with the maximum (minimum) MOKE signal. This is easily done knowing the MOKE signal of the initial magnetic configuration. This was done for each laser power and each magnetic configuration.

The following reasoning was first used by Liu[1]. Knowing the power P of the laser, the measured pulse energy $\bar{E}$ is found using the repetition rate f of the laser:

$$\bar{E} = \frac{P}{f} \#(S1)$$

The measured fluence $\bar{F}$ of the pulse is determined from the laser beam radius R, which is defined such that the power of the laser is divided by a factor e^2 at a distance R from the center of the beam:

$$\bar{F} = \frac{P}{f\pi R^2} \#(S2)$$

The power density profile I must then have the form:

$$I(r) = I_0 e^{-2\left(\frac{r}{R}\right)^2} \#(S3)$$

Where I_0 is the peak value of the power per unit area. The measured power must be equal to the surface integral of this function. One then finds:

$$I_0 = \frac{2P}{\pi R^2} \#(S4)$$

The fluence profile has then the form:

$$F(r) = 2\bar{F}e^{-2\left(\frac{r}{R}\right)^2} \#(S5)$$

The peak value of the fluence is then twice its measured value. A magnetic domain starts to appear as soon as the fluence peak value exceeds a certain threshold value F_{th}. When this happens, one would measure a fluence $\bar{F}_{th} = F_{th}/2$. The radius r of the domain must then verify the following equation:

$$F(r) = F_{th} \Leftrightarrow r = R\sqrt{\frac{1}{2}\ln\left(\frac{\bar{F}}{\bar{F}_{th}}\right)} \#(S6)$$

The fits in Figure 4 were done using the equivalent formula ($d = 2r$ and $D = 2R$):

$$d = D\sqrt{\frac{1}{2}\ln\left(\frac{\bar{E}}{\bar{E}_{th}}\right)} \#(S7)$$

We assume the laser beam diameter does not change during the measurements for one sample. Then all sets of data points for one sample are fitted with a common D i.e. the same D is assumed for all four magnetic configurations. Two sets of data points corresponding to magnetic configurations that are the time reversal symmetric of one another were also assumed to share the same $\bar{E}_{th}$. Data points were weighting by $1/\sigma^2$ where σ is the magnetic domain diameter uncertainty. Knowing $\bar{E}_{th}$ and D, one can calculate threshold fluences and the corresponding uncertainties with:

$$\bar{F}_{th} = \frac{4\bar{E}_{th}}{\pi D^2} \#(S8)$$

$$\mathrm{d}\bar{F}_{th} = \frac{4\ \mathrm{d}\bar{E}_{th}}{\pi D^2} + \frac{8\bar{E}_{th}\ \mathrm{d}D}{\pi D^3} \#(S9)$$

Where $\mathrm{d}\bar{E}_{th}$ is the measured pulse energy threshold uncertainty obtained from the data point fit and $\mathrm{d}D$ is the beam diameter uncertainty.

S5. Single layers

The hysteresis loops and magnetic behaviors after single laser pulse excitations of samples with a single layer of GdFeCo are shown in **Figure S1 and S2** for Gd concentrations x of 33% and 27.3% respectively. One can see that both samples exhibit a perpendicular magnetic anisotropy. When x = 33%, the GdFeCo alloy is Gd dominant (inverted hysteresis loop) and it does not exhibit AOS (white areas correspond to domain motion during image acquisition). When x = 27.3%, the sample is TM dominant with a higher coercive field because it has a composition quite close to the magnetic compensation composition (estimated to be around 27% in these deposition conditions). It exhibits AO-HIS. The coercive field is higher than in the hysteresis loop shown in the main text. This is because close to the magnetic compensation composition, slight changes of composition can lead to big changes of coercive fields[2].

S6. Absorption profiles

Absorption profiles calculated when a monochromatic plane wave with an 800 nm wavelength is sent directly (normal incidence) on the sample (top side excitation) or through the substrate (bottom side excitation) are shown in **Figure S3**. They were calculated using the Transfer Matrix Method[3]. For a top side laser excitation, we found that 7.92% of the light is absorbed by GdFeCo and 2.32% is absorbed by Co/Pt.

For a bottom side laser excitation, we found that 5.81% of the light is absorbed by GdFeCo and 6.50% is absorbed by Co/Pt.

Note also the difference of absorption in the Pt (4 nm) layer next to the Co/Pt multilayer in both cases.

We used the optical indices shown in **Table S2**. No change of optical index could be observed in the ellipsometry measurements for GdFeCo when the composition was changed. For the calculations in Figure S3, we used the common optical index value found in reference [4].

S7. High fluence behavior

The results obtained when exciting the sample with x = 33% with a fluence of 5.8 mJ cm^{-2} (pulse energy of 0.55 µJ) are shown in **Figure S4**. The same qualitative behavior than for lower fluences is observed except that a multidomain state appears in the center of the irradiation area. This multidomain state results from the dipolar field and thermal fluctuations generated by the strong heating in this area where the spin temperature approaches or exceeds the Curie temperature of one layer[5]. We can see that four different magnetic contrasts can coexist in this region. This supports the fact that any magnetic coupling between both layers is negligible.

S8. Top side laser excitation

The results obtained when exciting both samples from the top side are shown in **Figure S5 and S6** respectively. The results shown in the main text are obtained with a bottom side excitation. The corresponding hysteresis loops, obtained when measuring the Magneto Optical Kerr Effect through the substrate, are also presented. A negative slope, corresponding to the Faraday effect of the glass substrate, has been subtracted from both hysteresis loops. One can see that both AP configurations have changed Kerr rotation signs compared to Figure 1. This is due to the fact that the 10 nm of Cu attenuate the signal of the GdFeCo instead of the Co/Pt multilayer in this configuration. This means that what used to be a light red color is now a light blue color and vice versa.

The same reversal behavior as in the main text is observed. We also observe AP+ domains at the rim of the central P- domain after the second laser pulse when x = 33%.

References

[1] J. M. Liu, *Opt. Lett.* **1982**, *7*, 196.

[2] Y. Xu, M. Deb, G. Malinowski, M. Hehn, W. Zhao, S. Mangin, *Adv. Mater.* **2017**, *29*, 1703474.

[3] S. J. Byrnes, **2016**, arXiv:1603.02720 [physics.comp-ph].

[4] W. R. Hendren, R. Atkinson, R. J. Pollard, I. W. Salter, C. D. Wright, W. W. Clegg, D. F. L. Jenkins, *J. Phys. Condens. Matter* **2003**, *15*, 1461.

[5] T. D. Cornelissen, R. Córdoba, B. Koopmans, *Appl. Phys. Lett.* **2016**, *108*, 142405.

[6] K. M. McPeak, S. V Jayanti, S. J. P. Kress, S. Meyer, S. Iotti, A. Rossinelli, D. J. Norris, *ACS Photonics* **2015**, *2*, 326.

[7] W. S. M. Werner, K. Glantschnig, C. Ambrosch-Draxl, *J. Phys. Chem. Ref. Data* **2009**, *38*, 1013.

[8] R. Atkinson, S. Pahirathan, I. W. Salter, P. J. Grundy, C. J. Tatnall, J. C. Lodder, Q. Meng, *J. Magn. Magn. Mater.* **1996**, *162*, 131.

[9] N. Bergeard, M. Hehn, S. Mangin, G. Lengaigne, F. Montaigne, M. L. M. Lalieu, B. Koopmans, G. Malinowski, *Phys. Rev. Lett.* **2016**, *117*, 147203.

Figure S1. a) Hysteresis loop and b) results of sending a femtosecond laser pulse (top side excitation) observed using a MOKE microscope, on the single GdFeCo layer sample with x = 33%. θ_K is the normalized Kerr rotation in arbitrary units. The white region corresponds to magnetic domain motion during the averaging. The pulse energy is 0.70 µJ. The scale bar in the last image is 30 µm long.

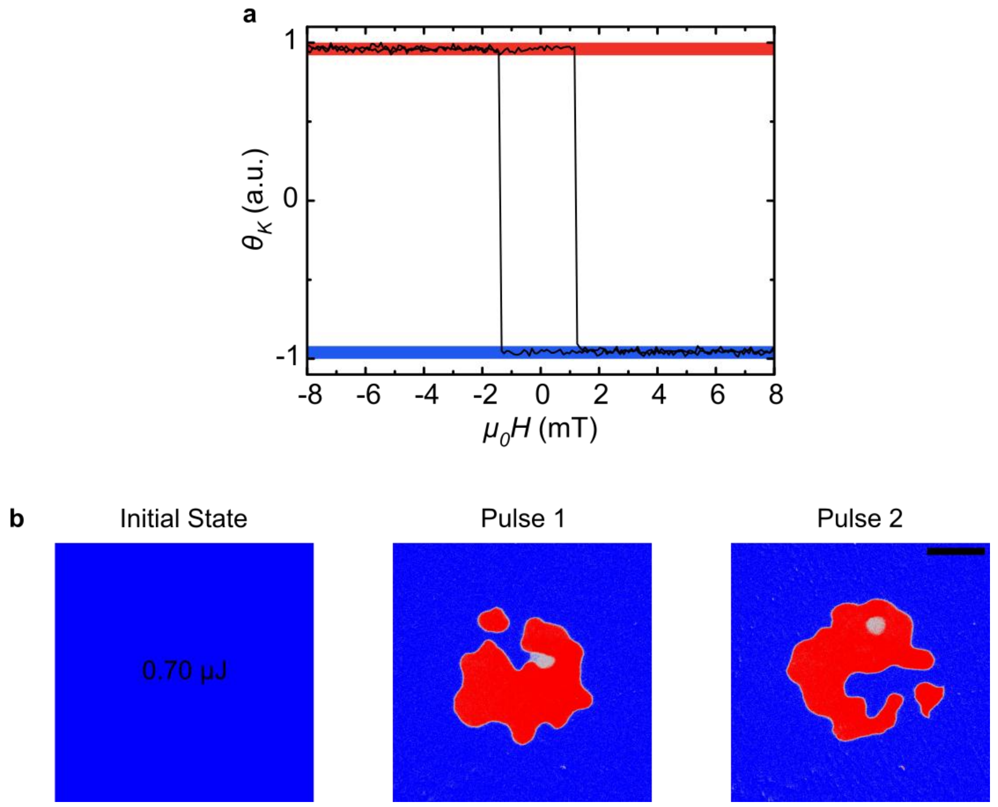

Figure S2. a) Hysteresis loop and b) results of sending a femtosecond laser pulse (top side excitation) observed using a MOKE microscope, on the single GdFeCo layer sample with x = 27.3%. θ_K is the normalized Kerr rotation in arbitrary units. The pulse energy is 0.09 µJ. The scale bar in the last image is 30 µm long.

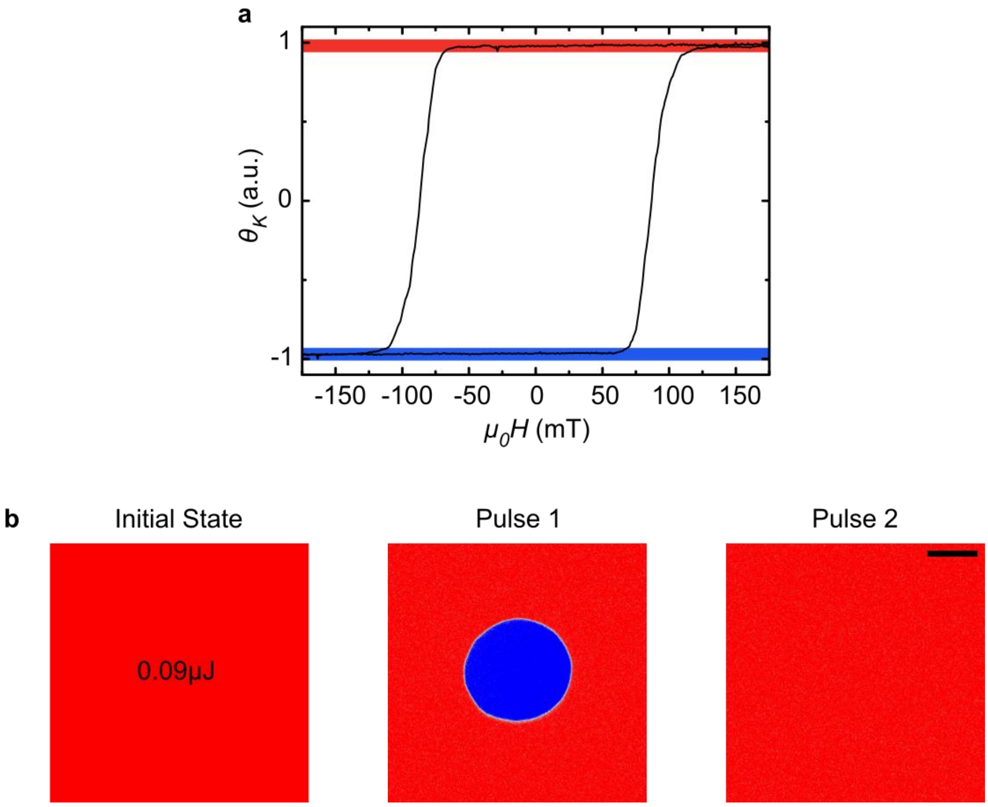

Figure S3. Absorption profiles for the spin valve samples as a function of the depth in the sample (starting from the top side surface) for a) top side or b) bottom side laser excitation.

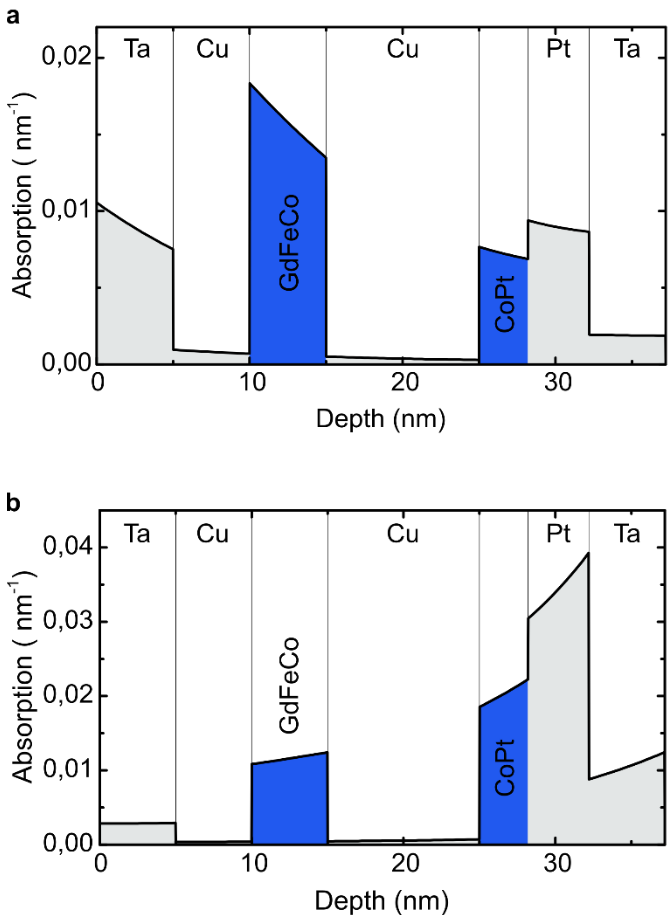

Figure S4. Results of sending a femtosecond laser pulse on the spin valve sample with x = 33% in a a) P+ or b) AP- configuration as observed using a MOKE microscope. The pulse energy is 0.55 µJ. The scale bar in the last image of each figure is 20 µm long.

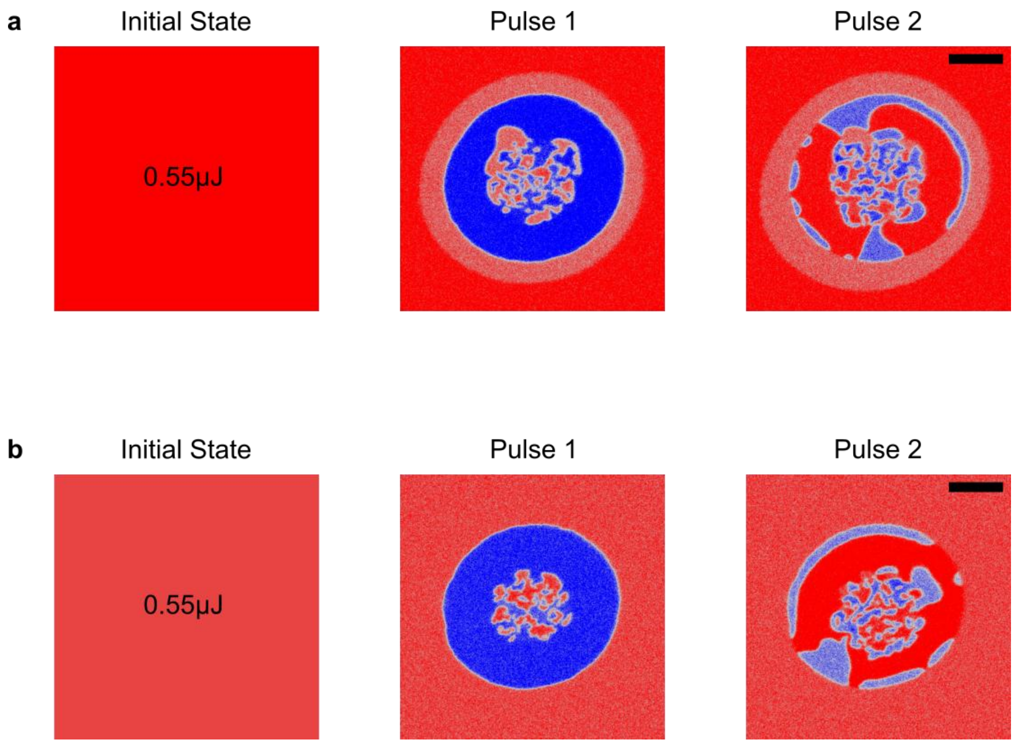

Figure S5. a) Hysteresis loop for the spin valve sample with x = 33% and bottom side observation. θ_K is the normalized Kerr rotation in arbitrary units. Results of sending a femtosecond laser pulse on the sample (top side excitation) are shown in a b) P+ or c) AP- configuration as observed using a MOKE microscope (bottom side observation). The pulse energy is 0.74 µJ. The scale bars in the last image of figure b) and c) are 30 µm long.

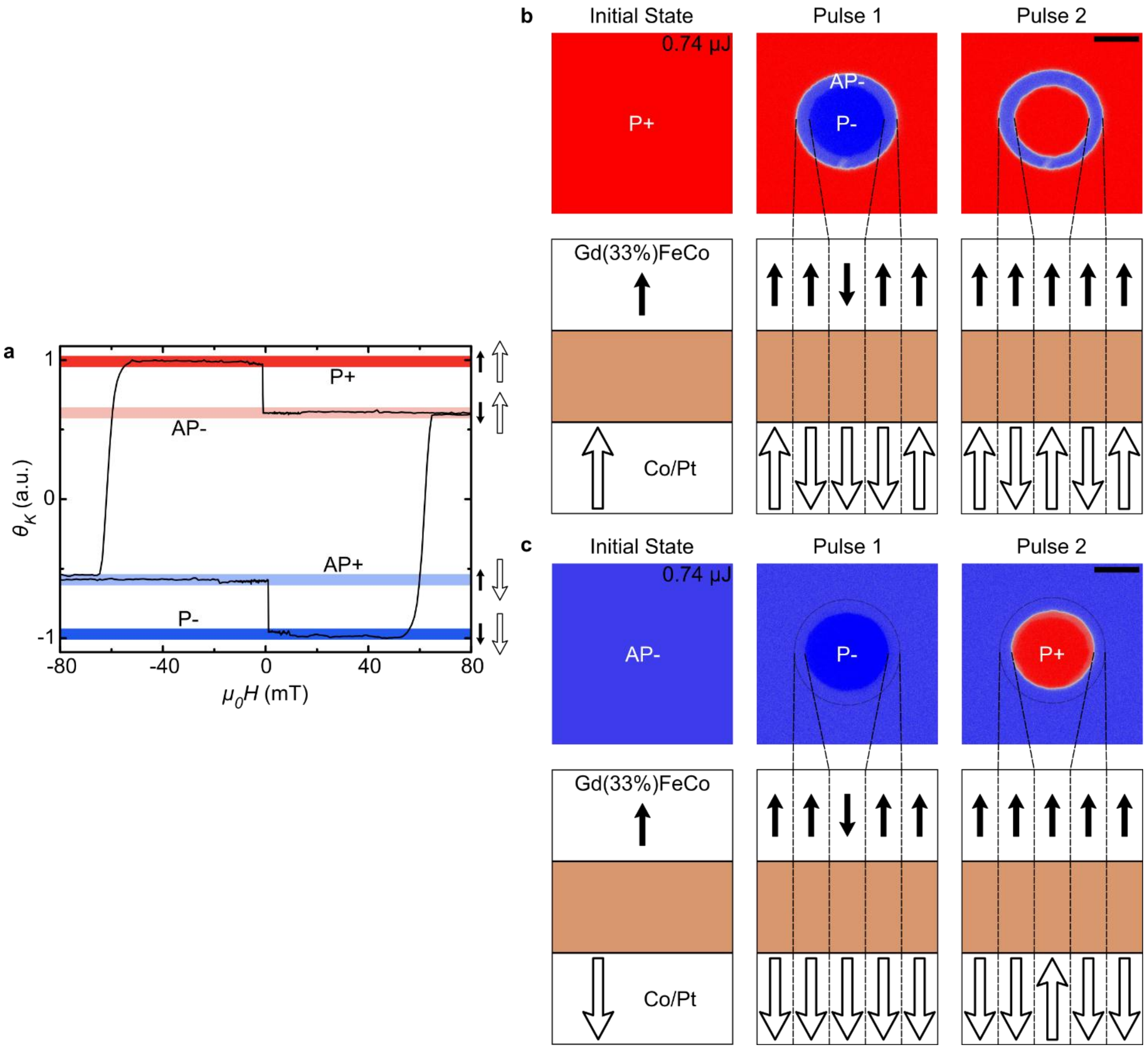

Figure S6. a) Hysteresis loop for the spin valve sample with x = 27.3% and bottom side observation. θ_K is the normalized Kerr rotation in arbitrary units. Results of sending a femtosecond laser pulse on the sample (top side excitation) are shown in a b) P+ or c) AP- configuration as observed using a MOKE microscope (bottom side observation). The pulse energy is 0.44 µJ. The scale bars in the last image of figure b) and c) are 30 µm long.

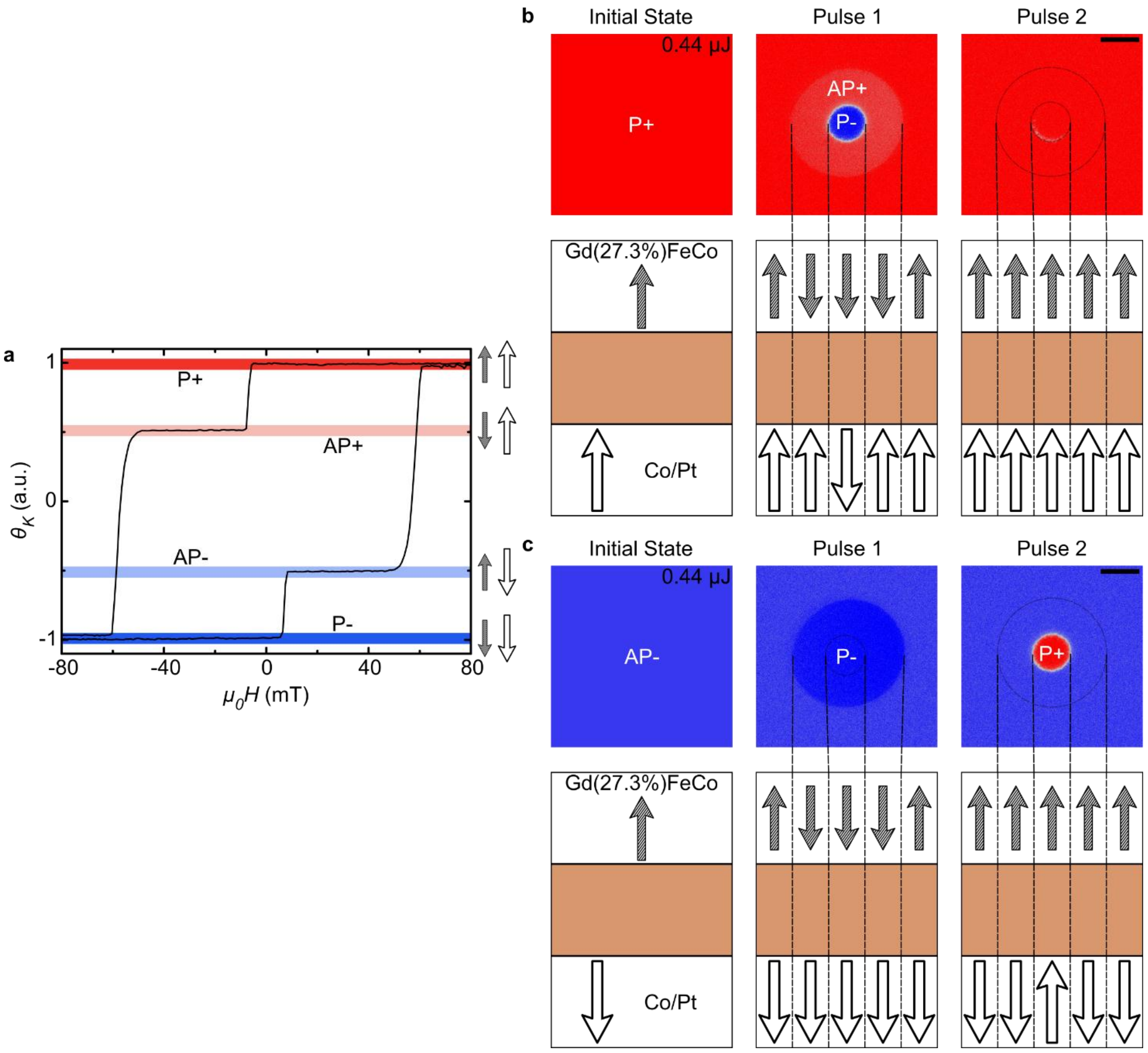

Table S1. Measured laser Beam diameters for both samples.

x	Beam diameter
27.3%	115 +/- 3 µm
33%	110 +/- 6 µm

Table S2. Optical indices of each layer.

Layer	n	Reference
Cu	0.10509+5.1413i	[6]
Pt	2.9779+6.3669i	DFT results in [7]
Ta	1.0057+4.2230i	DFT results in [7]
Co/Pt	2.6732+5.1883i	[8]
GdFeCo	3.7+3.855i	[4]
Glass	1.5136	[9]
